# To Remove Rust

**Published:** 1883-08

**Authors:** 


### TO REMOVE RUST.

To remove rust from steel instruments, rub them well with oil and let it lay for a few days. After that scour with quick-lime, when the rust will quickly disappear.—*Progrès Dentaire.*

